# Prediction of various freshness indicators in fish fillets by one multispectral imaging system

**DOI:** 10.1038/s41598-019-51264-z

**Published:** 2019-10-11

**Authors:** Sara Khoshnoudi-Nia, Marzieh Moosavi-Nasab

**Affiliations:** 1 Seafood Processing Research Group, School of Agriculture, Shiraz University, PO Box: 71441-65186, Shiraz, Iran; 2 Seafood Processing Research Group & Department of Food Science and Technology, School of Agriculture, Shiraz University, PO Box: 71441-65186, Shiraz, Iran

**Keywords:** Computational models, Quality of life, Risk factors

## Abstract

In current study, a simple multispectral imaging (430–1010 nm) system along with linear and non-linear regressions were used to assess the various fish spoilage indicators during 12 days storage at 4 ± 2 °C. The indicators included Total-Volatile Basic Nitrogen (TVB-N) and Psychrotrophic Plate Count (PPC) and sensory score in fish fillets. immediately, after hyperspectral imaging, the reference values (TVB-N, PPC and sensory score) of samples were obtained by traditional method. To simplify the calibration models, nine optimal wavelengths were selected by genetic algorithm. The prediction performance of various chemometric models including partial least-squares regression (PLSR), multiple-linear regression (MLR), least-squares support vector machine (LS-SVM) and back-propagation artificial neural network (BP-ANN) were compared. All models showed acceptable performance for simultaneous predicting of PPC, TVB-N and sensory score (R^2^_P_ ≥ 0.853 and RPD ≥ 2.603). Non-linear models were considered better quantitative model to predict all of three freshness indicators in fish fillets. Among the three spoilage indices, the best predictive power was obtained for PPC value and the weakest one was acquired for TVB-N content prediction. The best model for prediction TVB-N (R^2^_p_ = 0.862; RMSEP = 3.542 and RPD = 2.678) and sensory score (R^2^_p_ = 0.912; RMSEP = 1.802 and RPD = 3.33) belonged to GA-LS-SVM and for prediction of PPC value was BP-ANN (R^2^_p_ = 0.921; RMSEP = 0.504 and RPD = 3.64). Therefore, developing multispectral imaging system based on LS-SVM model seems to be suitable for simultaneous prediction of all three indicators (R^2^_P_ > 0.862 and RPD > 2.678). Further studies needed to improve the accuracy and applicability of HSI system for predicting freshness of rainbow-trout fish.

## Introduction

Consumption of fish, as an excellent source of omega-3 fatty acids, proteins and vitamins, is increased among people and became an essential part of a balanced human diet^1^. However, Fish quality deteriorates rapidly during post mortem storage. Given the importance of quality and safety assurance, this issue has received much attention and emphasis from the food industry practitioners, government and public^2^.

Hyperspectral imaging (HSI), as a non-destructive and rapid technique, exhibits its superiority to evaluate food quality and safety^2–4^. This method has already been used to assess the various fish freshness indicator, such as Trimethylamine (TMA), Total Volatile Basic Nitrogen (TVB-N)^5,6^, Thiobarbituric acid reactive substances (TBARS)^7^, total viable count (TVC)^8–10^, sensory factors^11,12^ and so on.

Psychrotrophic microbes can grow at low temperatures (below 5 °C). Therefore, they are greatly distributed in different refrigerated foods such as seafood^13^. This group of microorganisms affect the food quality by secreting the extracellular enzymes (e.g. lipase and protease) and consequently decrease the shelf-life of meat and seafood products^14^. Hence, the evaluation of these microorganisms can be especially importance for ensuring food safety and quality of refrigerated foods. However, the use of traditional method (plate count agar) for detection and measurement of psychrotrophic bacteria take a long time (incubation time is normally 7–10 days at 4–7 °C). Hence, it is necessary to develop rapid detection methods for psychrotrophic microorganisms^15^. In this regard, Barbin *et al*.^16^ determined the psychrotrophic plate count (PPC) in chilled pork during storage using near-infrared hyperspectral imaging (900–1700 nm) and the results showed that the great potential of HSI method for detecting this bacterial contamination in pork^16^. However, based on our best knowledge, no study focused on the application of HIS (400–1000) for evaluation of PPC in seafood. Therefore, in current study, for the first time, the application of HSI method was used to measure PPC in refrigerated rainbow trout fish fillets is being examined.

On the other hand, due to the complexity of quality deterioration in fish flesh, relying on a single freshness indicator cannot provide a reliable prediction from fish quality and safety^17^. Thus, the evaluation of quality and shelf-life in meat and seafood products could be more accurate when combined chemical, microbiological and sensory parameters are considered. In recent years, several attempts to develop a multispectral imaging system for predicting several spoilage parameters simultaneously. Cheng *et al*.^18^ developed a multispectral imaging method to predict various chemical spoilage in grass carp fillet^18^. Also, Siripatrawan (2018) developed a rapid and non-destractive method to assess the physicochemical, microbiological and sensory properties of dry cured sausage based on hyperspectral imaging and PLSR (partial least-squares regression)^19^. In current study, for enhancing the quality prediction performance, a simple multispectral imaging system was developed to evaluate chemical, microbial and sensory score of fish fillets, simultaneously.

Overall, developing a multispectral imaging system to evaluate the chemical, microbial and sensory quality of rainbow trout fish fillet during cold storage and comparison the prediction power of various linear (PLSR, multiple-linear regression: MLR) and non-linear (least-squares support vector machines: LS-SVM and back-propagation artificial neural network: BP-ANN) to evaluate various freshness factors (TVB-N, PPC and sensory score) and visualization of freshness indicators as concentration images are three of the main objects of current study.

## Results and Discussion

### References values analysis

**Figure 1:**
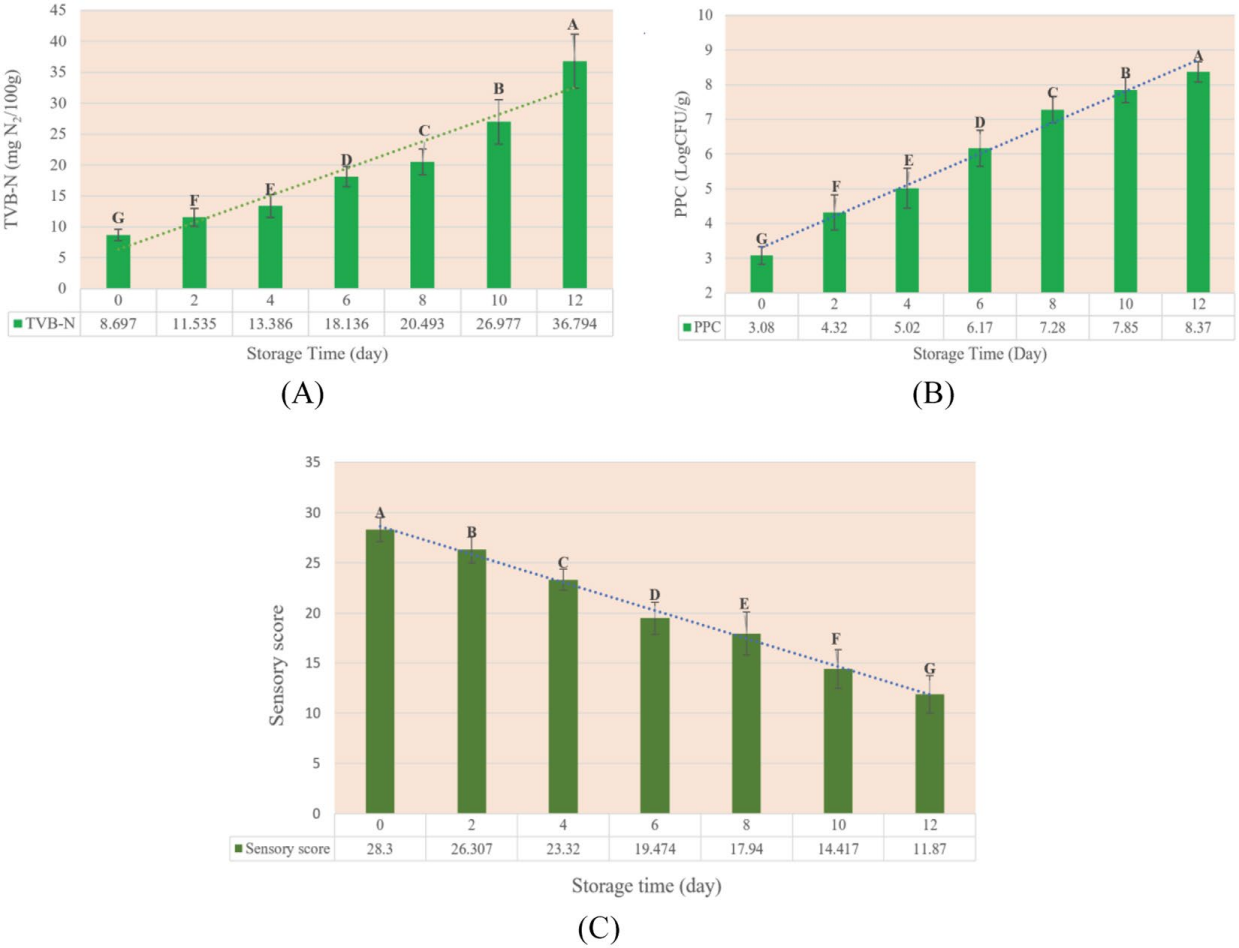
Changes of (**A**) TVB-N content, (**B**) PPC value and (**C**) sensory scores in rainbow trout fillets during 12 days storage at 4 ± 2 °C. Different letters indicate significant differences (*p* < 0.05).

Changes in sensory score, PPC and TVB-N values in 210 rainbow trout fillets during 12 days of cold storage were illustrated in Fig. 1A–C. Overall, Endogenous enzymatic and microbial activities are the most important reasons for postmortem degradation of fish fillets and consequently, decreasing the sensory acceptance of them^20^.

#### Analysis of TVB-N content

The initial TVB-N value of samples was 8.7 ± 0.9 mg N/100 g, which is comparable with those reported by previous authors for rainbow trout fish fillets^21–23^. During storage, this value was increased to 36.8 ± 4.4 mg N/100 g. If the acceptable level of TVB-N values in fresh fish was 20 mg N/100 g^24,25^, the shelf life of rainbow trout fish fillets was 8 days at 4 °C. Furthermore, a wide range of input data is an important factor to build an accurate, stable and robust calibration models^26^. The TVB-N value showed the variation range of 38.8 (7.31–46.11 mg N/100 g) and 35.46 (7.65 and 43.11 mg N/100 g) for calibration and prediction set, respectively, which were suitable range for establishing a robust prediction model.

#### Analysis of PPC value

Psychrotrophic microbes by secreting the various enzymes were considered as the most important responsible for the quality deterioration of seafood^13^. For this reason, the PPC value, as a significant freshness indicator, was evaluated in current study. The initial PPC value of the rainbow trout fillets was 3.08 ± 0.26 log_10_CFU/g, which increased with increasing storage time and finally reached to 8.37 ± 0.29 log_10_CFU/g at 12^th^ day. The initial PPC was similar to that reported by Jouki *et al*.^17^ and Pezeshk *et al*. in 2017 (3.1 and 3.48 log_10_CFU/g, respectively) and higher than that reported by Tabatabaei Moradi *et al*. in 2015 (2.11 log_10_CFU/g), for rainbow-trout fish^17,27,28^. These difference may be due to the type of nutrition, gender, fish life environment, fishing season and fish size^17^. The limit of acceptability for the PPC of fish is considered 7 log1_0_CFU/g^17^. Based on this threshold, the samples were unacceptable after 8 days cold storage. Furthermore, the variation range of PPC for calibration and prediction set were 6.14 (2.74–8.88) and 5.29 (2.82–8.74) log_10_CFU/g, respectively which are wide enough to establish a suitable model for prediction PPC in rainbow trout fish fillets.

#### Sensory analysis

The shelf life of fish, like other foods, highly related to consumer sensory acceptability^29,30^. The results of the sensory score of rainbow trout fillet samples are presented in Fig. 1C. Similar to other indicators, time had a significant effect on this factor (*p* < 0.05) and sensory score exhibited a decline trend with increasing storage time, so that within 8 days the total sensory score reached from 28.3 to below the acceptable threshold (<18: 17.94). Therefore, based on sensory analysis, shelf life was nearly 8 days. The results were consistent with previous findings for TVB-N and PPC factor and the correlation between TVB-N and sensory score and between PPC and sensory score was negative and strong (Pearson correlation: −0.933 and −0.918, respectively). Shi *et al*.^31^ also reported that the sensory analysis of tilapia fillets correlates well with the chemical and especially microbial analysis. In addition, variation range of sensory score for calibration set was 18.9 (10.2–29.1) and for prediction set was 18.6 (10.6–29.0).

### Spectral feature analysis

**Figure 2:**
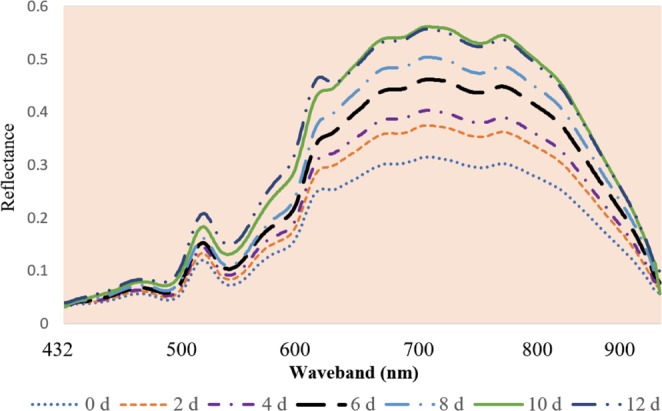
Average spectral features of rainbow trout fillets during cold storage. The abbreviation ‘d’ stands for day.

Near infrared spectroscopy (NIR-Spectroscopy) is a technology based on absorption of light by molecules^32^. Therefore, this technology can be provided the good information about chemical composition and bonding situation in fish flesh. Figure 2 illustrated the reflectance spectra plot of fish fillet based on different storage time intervals. As shown in this Fig. 2, with the passage time, the magnitude of spectral reflectance curves was increased. Probably, undecomposed chemical components of fish flesh have a lower reflectance than decomposed components producing during storage time^33^. Also, the several obvious absorption bands were recognizable on the spectra plot of samples. In general, the bands in visible range, 400–700 nm, (e.g. ∼440, 500, 560, 600 and 650 nm) probably related to the absorption of pigments of fish flesh. The peak of in near infrared range (700–1000 nm) may be connected to overtones of various chemical bonds, such as C-O, C-H, O-H, N-H and S-H stretching relating to products of lipid oxidation, protein and protein degradation products, sulphomyoglobin, water and so on^10,11,18,34^.

### Optimal wavelength selection

The elimination of redundant wavebands from the spectral set is a necessary step to establish a simple, less expensive and fast prediction model which can be suitable for on/in-line monitoring of food safety and quality^35^. In current study, genetic algorithm was used to select the most informative wavelength variables related to the fish quality from full spectral range. Nine wavelengths (488, 542, 576, 602, 626, 706, 764, 857 and 951 nm) were selected as the optimal predictors. These optimal wavelengths almost covered the whole spectral range. However, more than half of these wavebands were located in the visible region of the spectrum (400–760 nm). These results were agreed with other studies predicting various fish quality indicators such as TBARS^36^, *K*-value^18,37^, microbial spoilage^8,10^ and TVB-N^6^ based on optimal wavebands. Several previous studies reported the same phenomenon for various freshness factors, such as and TBARS^7^ in various fish fillets. The chemical, physical, microbial and enzymatic reaction occurring during storage can be affected the color of fish muscle that caused the spectral changes in visible region^36^. An optimal wavelength around at 550 nm can be connected to the stretching overtones of C–O and O–H bonds of protein. One of the most important selected wavelength located at ~630 nm was ascribed to the sulfhemoglobin absorption band resulting from the reaction of myoglobin and bacterial-produced H_2_S; a peak around 764 nm can be assigned to O–H stretching third overtone of water^38^. A peak at around 870 nm can be related to the vibrations of C–H and N–H possibly associating with protein, methylene group of lipid and other organic compositions^39,40^. Since, water in the fish flesh is the major component and finally, an optimal wavelength located at 950 nm was attributed to the water absorption band^8,41^.

### Comparison different chemometric models

In this work, hyperspectral imaging (HSI) coupled with various chemometric analysis including two linear (PLSR and MLR) besides two non-linear (LS-SVM and BP-ANN) have been used to estimate the major quality and safety indicators (e.g. TVB-N, PPC and sensory score) in rainbow-trout fish fillets. Comparison of the performance of linear and non-linear models in three individual sets (i.e. the calibration, cross-validation and prediction set) for prediction of PPC, TVB-N and sensory score were summarized in Table 1.

**Table 1:** Calibration, cross-validation and prediction results of the PPC, TVB-N values and sensory score of rainbow-trout samples by hyperspectral imaging system.

Model	n	LVs	Calibration		Cross-validation		Prediction			RDP
			R^2^_C(adj)_	RSMEC	R^2^_CV(adj)_	RSMECV	R^2^_P(adj)_	RSMEP	Bias	
**PPC (Log**_**10**_ **CFU/g)**										
PLSR	9	6	0.923	0.504	0.901	0.548	0.899	0.579	0.077	3.17
MLR	9	—	0.923	0.522	0.908	0.551	0.918	0.533	0.106	3.43
LS-SVM	9	—	0.920	0.515	0.904	0.505	0.917	0.517	0.103	3.55
BP-ANN	9	—	0.922	0.508	909	0.550	0.921	0.504	0.128	3.64
**TVB-N (mg N/100 g)**										
PLSR	9	5	0.889	3.084	0.874	3.304	0.857	3.585	0.257	2.645
MLR	9	—	0.892	3.164	0.870	3.355	0.855	3.593	0.174	2.640
LS-SVM	9	—	0.889	3.101	0.872	3.327	0.862	3.542	−0.208	2.678
BP-ANN	9	—	0.881	3.307	0.862	3.526	0.853	3.643	0.073	2.603
**Sensory score (6–30)**										
PLSR	9	4	0.927	1.572	0.919	1.604	0.902	1.987	−1.009	3.024
MLR	9	—	0.930	1.544	0.918	1.609	0.909	1.991	−0.983	3.018
LS-SVM	9	—	0.928	1.523	0.921	1.599	0.912	1.802	−0.996	3.335
BP-ANN	9	—	0.920	1.597	0.913	1.664	0.910	1.848	**−1.001**	**3.251**

LV: latent variable; R^2^_C(adj)_: adjusted determination coefficient of calibration; R^2^_CV (adj)_: adjusted determination coefficient of cross-validation; R^2^_P(adj)_: adjusted determination coefficient of prediction; RMSEC: root-mean-square errors estimated by calibration; RMSECV: root-mean-square errors estimated by cross-validation; RMSEP: root-mean-square errors estimated by prediction; MLR: Multi-linear regression; PLSR: partial least squares regression; LS-SVM: least squares support vector machine BP-ANN: back-propagation artificial neural network.

#### Prediction of TVB-N value

The comparison of prediction accuracy of four models showed that all of the chemometric models exhibited acceptable performance in prediction of TVB-N value (0.82 < R^2^_p_ < 0.9 and 2.5 < RDP < 3). Although, in calibration and cross validation set the performance of linear models were a little better than non-linear regression, but in the prediction set the best performance belonged to the LS-SVM model (R^2^_P_ = 0.862, RSMEP = 3.643 mgN/100 g and RDP = 2.678). In this regard, Cheng *et al*.^6^ reported that the prediction power of LS-SVM model for predicting TVB-N value was more than PLSR model (R^2^_P_ = 0.902 and 0.891; RMSEP = 2.782 and 2.807 respectively) in grass carp fish fillet^6^. Also, the prediction capability of LS-SVM (R^2^_P_ = 0.931 and RMSEP = 1.065 and RPD = 3.839) was evaluated higher than MLR (R^2^_P_ = 0.921 and RMSEP = 1.11 and RPD = 3.828) model by Cheng *et al*.^18^. Overall, Based on R^2^_P_, RMSEP and RDP, the performance of various chemometric models for prediction of TVB-N can be ordered from strongest to weakest as follows: LS-SVM > PLSR > MLR > BP-ANN. However, based on difference between RMSEP and RMSEC the best model was BP-ANN (RMSEP-RMSEC = 0.326 mg N/100 g). Furthermore, the lowest bias belonged to BP-ANN model. Bias value in LS-SVM model implies some under-fitting in predicted value but for other models, some over-fitting were obvious. It is worth mentioning, lower bias value does not necessarily mean superior model. Because the overfitted and underfitted values may neutralize each other.

Since the difference between performances of various models is little, it does not certainly state which models actually better than other. Overall, both linear and non-linear models can exhibit their merits. Linear models is faster, more simple and need relatively lower data, on the other hand non- linear models is more flexible, covers more data points and at the same time more complicated and demands more data to converge^42^. Therefore, depending on the situations and purpose both models can be applicable.

#### Prediction of PPC value

As shown in Table 1, although, the performance of linear and non-linear models for predicting of PPC value were very good/excellent but the predictive capability of nonlinear models were more satisfactory than linear regression. The non-linear model has a more flexibility and may be more suitable to handle the complexity of the relationship between a freshness index and the hyperspectral imaging data^42^. MLR, LS-SVM and BP-ANN models exhibited an excellent performance in prediction of PPC (R^2^_P_ > 0.9) and RPD index in all models was more than three. The best performance for predicting PPC was observed with R^2^_P(adj)_ = 0.921, RSMEP = 0.504 Log_10_CFU/g and RPD = 3.64 for BP-ANN model. There was no study on the PPC prediction in fish fillets by HSI system (400–1000 nm). However, Barbin *et al*.^16^ determined the psychrotrophic plate count in porcine meat during cold storage using near-infrared hyperspectral imaging (900–1700 nm) and PLSR model. They used regression coefficient to choose the optimal wavebands for prediction TVC and PPC values. The performance of RC-PLSR models for predicting TVC and PPC were relatively good (R^2^_p(TVC)_ = 0.81 and SEP_(TVC)_ = 1.0; R^2^_p(PPC)_ = 0.81 and SEP_(TVC)_ = 1.5). Furthermore, the predictive accuracy obtained in current study was better than those provided for TVC value in rainbow-trout fish fillets (RC-PLSR: R^2^_p_ = 0.866 and RMSEP = 0.781, n_sample_ = 108)^10^. Wu and Sun (2013) and Cheng and Sun (2015) reported excellent capability for TVC prediction in salmon (Competitive adaptive reweighted sampling (CARS)- PLSR: R^2^_P_ = 0.958 and RMSEP = 0.280; CARS-LS-SVM: R^2^_P_ = 0.967 and RMSEP = 0.265; n_sample_ = 60; n_sample_ = 60) and grass carp fish (Successive projection algorithm (SPA)-PLSR: R^2^_P_ = 0.9, RMSEP_(PLSR)_ = 0.57; SPA-LS-SVM: R^2^_P_ = 0.92 and RMSEP = 0.53; n_sample_ = 120), respectively^8,9^. Difference in chemometric analysis, waveband region range, freshness indicator, sample numbers, type of samples can be some possible reason for different results obtaining in various studies.

#### Sensory analysis

Seafood spoilage is a results of a sequence of changes which are perceivable by the human senses. Therefore, sensory evaluation is a scientific analysis to estimate the quality of seafood^43^. However, sensory analysis is subjective, time-consuming, destructive and not practical for large-scale^44^. Therefore, HSI method can be a good response to this problem. According to Table 1, based on RPD and R^2^_P_ parameters, all chemometric models showed excellent/ very good prediction power (R^2^_p_ > 0.9 and RPD > 3). However, the performance of non-linear regression models were little better than linear ones for prediction of sensory score. The best and weakest models for prediction of sensory score in rainbow trout were LS-SVM (R^2^_P_ = 0.912, RMSEP = 1.802 and RDP = 3.335) and PLSR (R^2^_P_ = 0.902, RMSEP = 1.987 and RDP = 3.024), respectively. Although, the difference between RMSEC and RMSEP for all models was low (<0.439) but the lowest one was obtained for BP-ANN model (0.251) that followed by LS-SVM model (0.279). This can be considered as another reason for more stability of the nonlinear model. Also, Cheng and Sun (2015), used hyperspectral imaging (400–1000 nm) in combination with LS-SVM model to predict sensory quality of grass carp fish fillet. They selected five optimal wavelength variables by successive projections algorithm (SPA). The predictive power of SPA-LS-SVM model was also very good/excellent (R^2^_P_ = 0.905, RMSEP = 0.922 and RDP = 3.01)^11^. Furthermore, bias value for all of models implies some under-fitting in sensory score predicted value.

In general, non-linear models were considered better quantitative model to predict all of the three freshness indicators in rainbow trout fish fillets. Several previous authors also reported that the capability of non-linear regression models was better than linear models for predicting various fish quality and safety indicators^5,9,37^. The complexity of the relationship between a freshness index and the data extracted from hypercube may be one of the possible reason for superiority of nonlinear model over linear one^36^.

Among the three spoilage indices, the best and weakest predictive power was obtained for PPC and TVB-N content prediction respectively. During storage time, produced volatile basic nitrogen formed from protein degradation can react with different fish compositions. Consequently, chemical factor can subject to many variations^17,45^. This reason make the prediction of chemical indicator such as TVB-N more complex. However, Shi *et al*.^31^ used optimal wavelengths from hyperspectral imaging (HSI) selected by successive projections algorithm (SPA) and radial basis function neural network to predict various freshness factor in tilapia fillets. Their results showed that the relative error of TVB-N, TVC and *K* value between predicted and experimental values were 5.66, 6.29 and −4.84%. In the other word the best prediction performance was obtained for K value and the weakest one was acquired for TVC value. Different chemometric analysis, fish Species, and experimental condition could explain the disagreement between various research findings^31^.

Overall, the best model for prediction TVB-N and sensory score was GA-LS-SVM model and for prediction of PPC value the best performance belonged to BP-ANN. Therefore, developing multispectral imaging system based on LS-SVM model seems to be more suitable for simultaneous prediction of all three indicators. As a result, the visualization procedure was performed based on simplified LS-SVM model.

### Distribution maps

**Figure 3:**
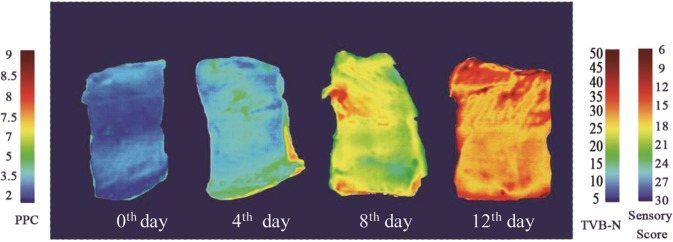
Distribution maps of freshness quality of rainbow-trout fillets stored at 4 °C for 12 days.

Visualization of the freshness indicators as a pseudocolor map is the final and the most important stage in hyperspectral imaging system. Such map in a pixel-wise manner is the main superiority of HSI over the traditional spectroscopy^5^. Figure 3 shows four examples of distribution maps of fish fillet samples at different storage times (0, 4, 8 and 12 days). In this Figure the changes of PPC, TVB-N values and sensory score is indicated sample by sample and even location by location in the same sample. A linear color scale shows the quality status in various location of fish flesh during storage time. In this color bar, blue and red color represents the best (low values of PPC, TVB-N and high sensory score) and the worst quality status of fish fillets, respectively. Due to the various speeds of chemical and microbial degradation of various compounds, the freshness and the quality of different location of a sample is not uniform. As a result, distribution of colors in the map is not homogeneous, indicating the various degree of TVB-N, PPC value and sensory score in different region of the same fish fillet. For example, in the first day of storage, most pixels are dark blue indicating high quality (the low PPC and TVB-N values and the high sensory score) of rainbow trout fish fillets. During the spoilage process, the color of pixels changed and finally at the last day of storage, most pixels change to the red color. As shown in Fig. 3 usually the edge of fish sample showed a low quality (yellow-red color). This demonstrated that the chemical and microbial spoilage progressed from surrounding area inner region of samples^46^. Because the external parts of fish fillets were more subjected to oxidation, microbial contamination and physical damage^47^.

## Materials and Methods

### Fish fillets preparation

Forty freshly killed rainbow-trout (*Oncorhynchus mykiss*) fish (0.75–1.2 kg) from the same batch were purchased from a local aquaculture pond (Bajgah, Shiraz, Fars, Iran). They immediately transported in insulated ice boxes to the Seafood-Processing Research Group laboratory (Shiraz, Iran). After rigor mortis and removing internal organs, the fish samples were beheaded, filleted, and finally washed with cold water. Fish fillets were cut into a size of 8.0 × 4.0 × 1.0 cm (length × width × thickness). Thus, 210 subsamples were obtained. All the subsamples were labelled, placed into plastic zip bags, and randomly divided into seven groups containing 30 subsamples. These groups were stored at 4 ± 2 °C for 0, 2, 4, 6, 8, 10 and 12 days to indicate the changes of fish muscle from freshness to spoilage. To exhibit the suitability of the model, 75% of subsamples were classify in training (calibration) group (140 subsamples) and 25% was used to test the model (prediction set: 70 subsamples). Furthermore, the classic Kennard–Stone (KS) algorithm was used to reduce bias in classification of samples into the calibration and prediction groups^48^.

### Acquisition and calibration hyperspectral images

The hyperspectral images were obtained by a Hyper Spectral Imaging (1000) spectrograph (Opt Co., Kashan, Iran). The spectrograph covered the waveband range of 400–1160 nm (spectral resolution: 2.5 nm, spot diameter less than 5 μm, the resulting images were of 600 × 400 pixels). However, due to the presence of too much noise in the spectral regions below 430 nm and over 1010 nm, the region of 430–1010 nm was considered for analysis. The illumination unit containing two daylight fluorescent (36 W) and two tungsten (100 W) lamps are located at two sides of the mobile platform. The speed of mobile platform was 0.6 mms^−1^ which is controlled by a computer system (*LabVIEW 2011*, National Instruments CO. Austin, USA). For imaging, a subsample was placed on the mobile platform stage and scanned line by line.

In order to minimize the effects of detector sensitivity, illumination and differences in physical and camera configuration of the imaging system current of camera, the raw format of hyperspectral images (R_S_) were calibrated into the reflectance mode with two extra images for dark (D: fully covering the camera lens and ~0% reflectance) and standard white (W: uniform Teflon white tile with ~99% reflectance) reference images, the calibrated image (R_C_) was estimated by the following formula^3^:

1 $${R}_{C}=\frac{{R}_{S}-{R}_{D}}{{R}_{W}-{R}_{D}}$$

where R_C_ is the intensity value of a raw hyperspectral image, R_D_ is the intensity value of dark image, and R_w_ is the intensity value of white reference image^3^.

### Determination of reference data set

Immediately after scanning, the samples were analyzed for PPC, TVB-N and Sensory score using the conventional methods as follow:

#### PPC value

The number of viable bacteria of each chopped sample was measured according to standard plate count agar (PCA Merck) based on ISO 8443:2003 method. Plates were incubated at 7 °C for 10 days^49^. The number of bacterial colonies were transformed into logarithms of the number of colony forming units (CFUs) per gram (log_10_ CFU/g).

#### TVB-N content

TVB-N content of rainbow trout was determined by the Kjeldahl distillation mechanism as described by Goulas and Kontominas (2005) and presented as mg N/100 g of fish sample^50^.

#### Sensory evaluation

The sensory quality of rainbow trout fillets were evaluated by a panel of 10 trained assessors based on the International Organization for Standardization method. The fillets were evaluated based on two criteria for raw (color, odor, texture) and cooked (odor, taste, texture) samples. Panelists scored for sensory characteristics using a five-point hedonic scale (from 1(very bad) to 5 (very good)). Sensory analysis was conducted light in the sensory laboratory and under cool white fluorescent^51,52^. A total score of 18 was considered as the threshold for sensory acceptable and under this score sample was rejected.

### Average-spectra extraction and pre-processing

After the acquisition hyperspectral images, the region of interests (ROIs) were identified by ROI tool in the ENVI v5.4 software (ITT Visual Information Solutions, Research Systems Inc., Boulder, CO, USA). Savitzky-Golay (S-G) smoothing technique using chemometric software Unscrambler 10.4 (CAMO, Trondheim, Norway) was applied to reduce the noises of the extracted average spectra.

### Optimal wavelength selection

The hyperspectral images of each fish fillet sample comprised of hundreds of contiguous wavelengths. The most of the wavelengths have a weakly related to the prediction of freshness indicator(s). Therefore, eliminating these useless variables is important to decrease the burden of data processing and obtain a more accurate, robust and simple model. In the current study a genetic algorithms (GA) was applied to choose the most informative spectral variables.

GA is a stochastic search and heuristic method including six steps: (1) randomly coding of all variables by 0 and 1; (2) initiating the population; (3) evaluating the responses by RMSECV; (4) reproducing the responses with high RMSECV; (5) mutations. (6) alternating steps three to five until achieving the predefined numbers of iterations and identifying the variables with the most frequent^53^.

### Chemometric analysis

The calibration between the spectral and the reference data (i.e., TVB-N, PPC and sensory score) were established by PLSR and MLR as a typical and robust linear modeling method. Furthermore, since the spectral data may be polluted by various nonlinear and ambiguous parameters such as stray light^54^. Thus, BP-ANN and LS-SVM models, as non-linear calibration models were also used for building prediction models. The linear models were carried out in Unscrambler 10.4x software (CAMO, Trondheim, Norway) and the non-linear regressions were performed in MATLAB R2016a (The Mathworks Inc., Natick, MA, USA).

### Model evaluation

The spectral data selected by GA were considered as input for establishing various linear and nonlinear models and the performance of them was compared. The assessment factors include the adjusted determination coefficient (R^2^_C(adj)_, R^2^_CV(adj)_, and R^2^_P(adj)_), the root mean square error of them (RMSEC, RMSECV and RMSEP), bias and residual predictive deviation (RDP). These coefficients were calculated by following equations:

2 $${R}^{2}=1-\frac{{\sum }^{}{({y}_{i,pred}-{y}_{i,act})}^{2}}{{\sum }^{}{({y}_{i,pred}-\bar{y})}^{2}}$$

3 $${R}_{Adj}^{2}=1-(1-{R}^{2})\frac{(m-1)}{m-p-1}$$

4 $$RMSE=\sqrt{\frac{1}{m}\mathop{\sum }\limits_{i=1}^{m}{({y}_{i,pred}-{y}_{i,act})}^{2}}$$

5 $$Bias=\frac{1}{m}{\sum }^{}({y}_{i,pred}-{y}_{i,act})$$

6 $$RPD=\frac{SD}{RMSEP}$$

*y*_*i*,*pred*_: the predicted value for a particular freshness indicator; *y*_*i*,*act*_: the measured (by tradition method) value for a particular freshness indicator; *m*: the number of samples and *p*: the number of predictors; SD: standard deviation of reference values^55^.

Generally, a suitable prediction model should have higher values of determination coefficient (R^2^ < 0.82: poor model; 0.82 ≤R^2^ ≤0.9: good model and R^2^ > 0.9: excellent model) and RPD (RPD < 1.5: very poor model- is not recommended; 1.5 < RPD < 2.0: poor model- only high and low values are distinguishable; 2 < RPD < 2.5: fair model- may be used for approximate quantitative predictions; 2.5 < RPD < 2.5: good model- quantitative predictions can be made with good prediction; RPD > 3: very good model- quantitative predictions can be made with very good prediction and R > 5: excellent model) and lower values of RMSEs as well as a small difference between RMSEC and RMSEP^2,56,57^.

The building, validation, and evaluation processes of PLSR and MLR models were carried out using Unscrambler 10.4x software (CAMO, Trondheim, Norway) and LS-SVM and BP-ANN models were performed in MATLAB R2016a (The Mathworks Inc., Natick, MA, USA).

### Visualization of freshness information

**Figure 4:**
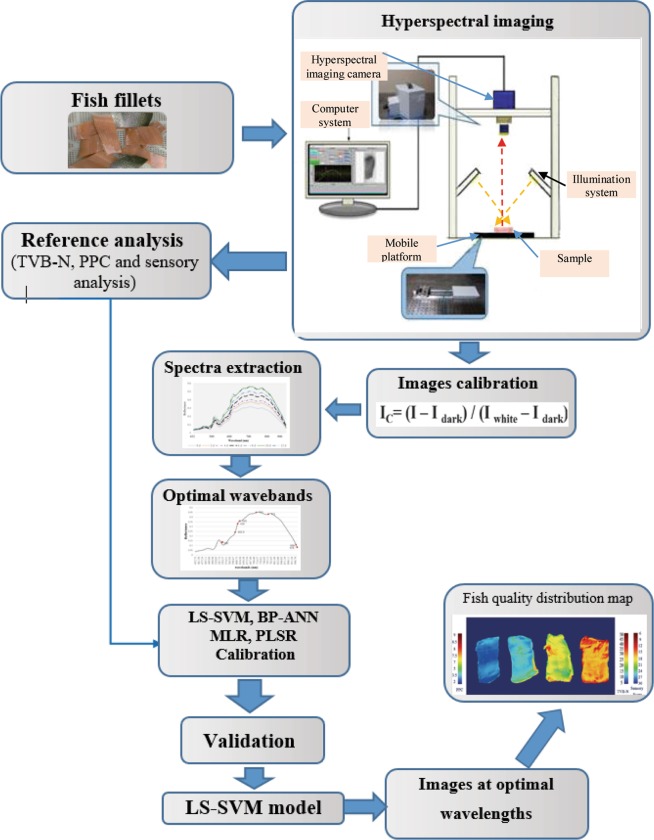
Flow chart for prediction of TVB-N, PPC values and sensory score in rainbow trout fish fillets by HSI technique (430–1010 nm).

One of the most important advantage of hyper/multispectral imaging system is visualization of the sample quality as distribution maps to clearly observe the degree of chemical, bacterial and sensory spoilage in the fish fillets from sample to sample and pixel to pixel. In the procedure, each pixel of image has an individual spectral profile and the optimized models (GA-LS-SVM) were used to transfer the spectrum of each pixel of sample ROI into the distribution map. Then, the predicted values were displayed with different colors and the pseudo color maps of PPC value, TVB-N content and sensory score were obtained to directly recognizing the quality of rainbow-trout fish flesh. A linear color bar was applied to exhibit the different values of the three quality parameters (blue and red color represent low and high value of each index, respectively)^58^. The visualization procedure was programmed in MATLAB R2016a software. Figure 4 illustrates the main steps for prediction of freshness indicators in rainbow trout fish fillets using hyperspectral imaging.

### Ethical approval

This article does not contain any studies with animals performed by any of the authors. This article does not contain any studies with human participants or animals performed by any of the authors.

## Conclusions

A hyperspectral imaging technique system (432–1010 nm) coupled with various linear (PLSR and MLR) and nonlinear (LS-SVM and BP-ANN) models were applied to evaluate PPC, TVB-N values and sensory score, as three essential fish freshness and quality indicators. In order to establish one multispectral imaging system for rapid determination of the quality indicators in fish flesh, nine optimal wavebands were selected by genetic algorithm. The results showed that the prediction performance of nonlinear models was better than nonlinear ones for evaluating freshness quality of rainbow trout fillets. The best predictive power was obtained for PPC value, followed by sensory score and TVB-N content. Due to evaluation of PPC value take a long time to complete (10 days incubation) the use of multispectral imaging method as a non-destructive and rapid method for estimation of this factor in fish fillets has a great advantage. Also, the use of LS-SVM model for prediction of TVB-N value and sensory score was superior over other chemometric models (R^2^_P_ = 0.912, RPD = 3.33 and R^2^_P_ = 0.862, RPD = 2.678 respectively). Furthermore, BP-ANN was the best chemometric model for prediction of PPC value in rainbow trout fish fillets (R^2^_P_ = 0.921, RPD = 3.64). As a result, the visualization procedure was carried out based on LS-SVM model. Overall, the current results showed that the use of multispectral imaging coupled with chemometric models, especially non-linear ones, is promising method to predict PPC, TVB-N content and sensory score in rainbow trout fish fillets and the technique can replace the tradition analyses method. However, further studies needed to improve the accuracy and applicability of HSI system for prediction freshness indicator of rainbow-trout fish by investigation on other nonlinear regression (such as Deep Learning Method) and/or variable selection methods. The Classification of fish samples in 3–5 quality class based on new models such as Multi-layer Extreme Learning Machine-based Autoencoder^59^, Spatial Prior Fuzziness Pool-Based Interactive classification^60^ and Sammon projection and wavelet kernel extreme learning machine^61^ can be proposed to probably improve the prediction power of HSI system.

## Data Availability

The datasets generated and/or analyzed during the current study are available from the corresponding author on reasonable request.
